# Intoxication au M’khinza: à propos de deux observations

**DOI:** 10.11604/pamj.2018.31.18.15987

**Published:** 2018-09-06

**Authors:** Luc Pascal Christian Loulouga Badinga, Nour Mekaoui, Lamiae Karboubi, Badr Sououd Benjelloun Dakhama

**Affiliations:** 1 Urgences Médicales Pédiatriques, Hôpital d’Enfants, Centre Hospitalier Ibn Sina, Rabat, Maroc; 2 Faculté de Médecine et de Pharmacie, Université Mohamed V, Souissi, Rabat, Maroc

**Keywords:** Poisoning, child, anserine, M’khinza, Intoxication, enfant, ansérine, M´khinza

## Abstract

Dysphania ambrosioides ou ansérine, appelée au Maroc M'Khinza est une plante appartenant à la famille des Chenopodiaceae. Utilisée au Maroc pour ses propriétés thérapeutiques notamment antipyrétique, elle peut être toxique si elle est mal dosée. Nous rapportons deux cas d'intoxications colligés au service des urgences médicales pédiatriques de Rabat: un nourrisson de 5 mois et une fille de 10 ans qui, suite à l'ingestion d'infusion de cette plante à des doses indéterminées dans un but antipyrétique avaient présenté respectivement une encéphalopathie toxique et une déshydratation sévère sur gastroentérite aiguë aboutissant aux décès en moins de 12 heures. Ces nouveaux cas de neurotoxicité et d'entérotoxicité au M'Khinza doivent interpeler sur la nécessité de savoir évoquer le diagnostic, d'informer, de lutter contre la banalisation de sa consommation, d'inciter à la recherche sur la pharmacopée traditionnelle permettant d'identifier leurs propriétés thérapeutiques afin de formaliser, rationaliser et codifier leurs prescriptions.

## Abstract (en)

Dysphania ambrosioides or anserine, known in Morocco as M’Khinza, is a plant belonging to family Chenopodiaceae. In Morocco, it is used for its therapeutic properties including, in particular, antipyretic properties. However, it can be toxic if it is incorrectly calibrated. We report two cases of poisoning whose data were collected in the Pediatric Emergency Department in Rabat. The study involved a 5-month old infant and a 10 year old girl with, respectively, toxic encephalopathy and severe dehydration associated with acute gastroenteritis after the ingestion of undetermined doses of infusion of this plant used as antipyretic. They both died in less than 12 hours. This study highlights the importance of considering M’Khinza-induced neurotoxicity and enterotoxicity in order to reach a correct diagnosis, the role of information and the need to fight against harmful consumption. Research on traditional pharmacopoeia should be encouraged in order to identify the therapeutic properties and to formalize, streamline and codify prescriptions.

## Introduction

**Figure 1:**
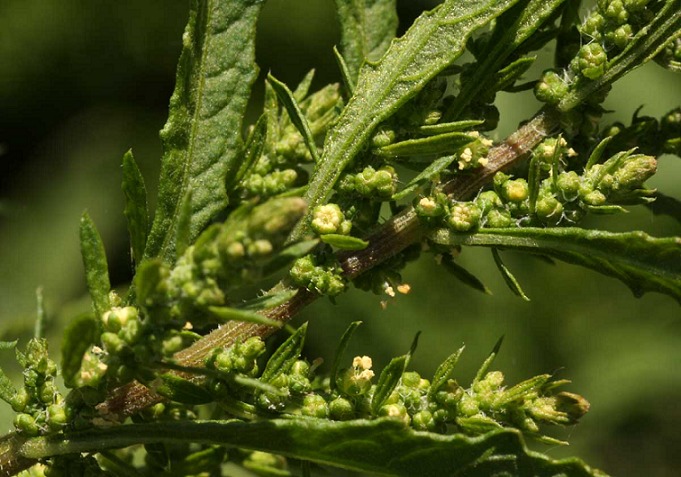
Dysphania ambrosioide

Autrefois nommée chenopodium ambrosioides, dysphania ambrosioides (Figure 1) ou ansérine, est une plante herbacée appartenant à la famille des Chenopodiaceae. Appelée M´Khinza au Maroc, elle est utilisée pour ses propriétés thérapeutiques par voie interne ou externe comme vermifuge, astringent, antispasmodique, carminative, antipyrétique ou comme cicatrisant contre les ulcérations buccales. Cependant, elle peut être facilement toxique si elle est mal dosée. Nous rapportons deux cas d'intoxications à la plante M'khinza colligés au service des Urgences Médicales Pédiatriques de Rabat: un nourrisson de 5 mois et une fille de 10 ans qui avaient présenté des complications suite à l'ingestion d'infusion de cette plante dans un but antipyrétique.

## Patient et observation

**Observation1:** Il s'agit d'une fille de 10 ans, admise aux urgences médicales pédiatriques en état de mal convulsif. Elle était suivie pour dilatation des bronches. Suite à une fièvre aiguë non chiffrée de moins de 24 heures sans autres signes associés, les parents avaient administré, la plante M'khinza en infusion en quantité importante et répétée. Une à deux heures après l'ingestion, l'évolution était marquée par une altération de la conscience suivie de crises convulsives tonicocloniques généralisées avec révulsion des yeux et émission d'une expectoration mousseuse, d'une durée d'environ deux heures, sans reprise de la conscience. Admise en crise convulsive, elle était apyrétique, polypnéique (fréquence respiratoire à 35 cycles/minutes) sans signes de lutte respiratoire, normocarde à 80 battements par minutes, une glycémie capillaire à 0,97 g/l et une pression artérielle à 10/6 cmHg. Le reste de l'examen somatique était sans particularité. Mise en condition, elle avait bénéficié d'une dose de charge de phénobarbital précédée de 0,5 mg/kg de diazépam en intrarectal responsables d'un arrêt des convulsions sans reprise de la conscience, aboutissant au décès une heure après l'admission au service de réanimation.

**Observation 2:** Il s'agit d'un nourrisson de 5 mois, de sexe masculin, admis aux urgences médicales pédiatriques en état de choc sur déshydratation aiguë sévère. Sans antécédent pathologique notable, il était unique de sa fratrie. Les parents rapportaient une fièvre non chiffrée évoluant depuis 10 jours motivant l'administration de la plante M'khinza au nourrisson cataplasme sur le front, puis par voie orale en quantité non précisée se traduisant par des vomissements incoercibles associés à une diarrhée liquidienne compliqués d'une déshydratation aiguë sévère avec refus de téter. L'examen clinique à l'admission trouvait un nourrisson hypotonique, peu réactif, une fontanelle antérieure déprimée et des yeux creux, des extrémités froides, une hypothermie a 36°C et un pli paresseux de déshydratation aboutissant au décès 10 heures plus tard au service de Réanimation.

## Discussion

Malgré les progrès de la pharmacologie, l'usage thérapeutique des plantes est très présent dans certains pays du monde surtout ceux en voie de développement nonobstant un système médical moderne. Le Maroc par la richesse et la diversité de sa flore, constitue un véritable réservoir phytogénétique avec environ 4500 espèces et sous espèces de plantes parmi lesquelles Dysphania ambrosioides (Figure 1) [1]. Dans la littérature, les données concernant l'intoxication à l'ansérine chez l'enfant, sont rares et peu documentées. En général, elles concernent les intoxications liées aux médicaments, aux produits ménagers, aux produits issus de la pharmacopée traditionnelles ou aux plantes. L'utilisation pour des fins alimentaires, curatives ou esthétiques de certaines plantes éventuellement toxiques, ou du moins une partie (graine, tige, etc.), peut induire de sérieuses intoxications, voire mortelle. Ces intoxications constituent un accident fréquent dans la plupart des régions du monde [2]. Dysphania ambrosioides (Figure 1) est une plante herbacée, annuelle ou vivace de 30 cm à 1 m de haut dégageant une odeur lorsqu'elle est froissée. Originaire d'Amérique centrale et du sud, elle est cultivée dans le Maryland (États-Unis) et en Chine. Au Maroc, elle pousse dans les lieux incultes, abandonnés et aux bords des chemins. Toutes ses parties (racines, feuilles, fleurs, écorces, graines) quelles soient fraiches ou desséchées sont utilisées à des fins thérapeutiques. Les principaux principes actifs d'extrait d'huile essentielle de ses feuilles sont le p-cymène (50,0 %), l'α-terpinène (37,6%) et l'ascaridol (3,5 %) [3, 4]. Ses propriétés thérapeutiques ont fait l'objet de plusieurs publications qui lui attribue une action antifongique [5], antiparasitaire [6], antibactérienne et antimycobactérienne [7], ou antirhumatismal [8]. Quelques cas de toxicité encéphalique [9] ou rénale [10] de ce principe actif chez l'enfant ont été rapportés dans la littérature. Concernant nos patients, suite à une tendance à majorer la dose ou à multiplier les prises lorsque l'effet escompté n'était pas atteint, ils avaient présenté respectivement des signes de toxicité neurologique et gastro-intestinale aboutissant à leurs décès en moins de 12 heures.

## Conclusion

Ces nouveaux cas de neurotoxicité et d'entérotoxicité à l'ansérine devraient interpeller: les cliniciens qui doivent pouvoir évoquer la possibilité d'intoxication à l'ansérine devant les éléments anamnestiques et cliniques précédemment décrits; sur la nécessité d'informer les praticiens et les consommateurs de plantes médicinales afin de lutter contre la banalisation de leur consommation; à la recherche sur la pharmacopée traditionnelle afin d'en identifier les propriétés thérapeutiques, de formaliser, de rationaliser et de codifier les prescriptions. Ceci permettra d'éviter les risques d'intoxication dont les conséquences peuvent être fatales.

## Conflits d’intérêts

Les auteurs ne déclarent aucun conflit d'intérêts.

## Contributions des auteurs

Tous les auteurs ont lu et approuvé la version finale du manuscrit.
